# A High-Grade Glioma, Not Elsewhere Classified in an Older Adult with Discordant Genetic and Epigenetic Analyses

**DOI:** 10.3390/biomedicines12092042

**Published:** 2024-09-08

**Authors:** Carlen A. Yuen, Silin Bao, Xiao-Tang Kong, Merryl Terry, Alexander Himstead, Michelle Zheng, Melike Pekmezci

**Affiliations:** 1 Neuro-Oncology Division, Department of Neurology, University of California, Irvine, CA 92868, USA; 2 Neurosciences Division, Department of Internal Medicine, Community Regional Medical Center, Fresno, CA 93721, USA; 3 Department of Pathology, University of California, San Francisco, CA 94143, USA; 4 Department of Neurosurgery, University of California, Irvine, CA 92868, USA; 5 UC Irvine Charlie Dunlop School of Biological Sciences, University of California, Irvine, CA 92697, USA

**Keywords:** high-grade astrocytoma with piloid features, HGAP, HGG NEC, pilocytic astrocytoma, DNA methylation, classification, glioblastoma, high-grade glioma, not elsewhere classified, WHO classification, central nervous system tumors

## Abstract

The World Health Organization’s (WHO) classification of central nervous system (CNS) tumors is continually being refined to improve the existing diagnostic criteria for high-grade gliomas (HGGs), including glioblastoma. In 2021, advances in molecular analyses and DNA methylation profiling were incorporated to expand upon the diagnostic criteria for HGG, including the introduction of high-grade astrocytoma with piloid features (HGAP), a new tumor entity for which a match to the HGAP class in DNA methylation profiling is an essential criterion. We present an equivocal case of a 72-year-old male with an HGG exhibiting features of both HGAP and glioblastoma, but which did not conform to any existing 2021 WHO classification of CNS tumor entities. This “no match” in DNA methylation profiling resulted in a final diagnosis of HGG not elsewhere classified (NEC), for which standard treatment options do not exist.

## 1. Introduction

The World Health Organization’s (WHO) classification of central nervous system (CNS) tumors has undergone multiple revisions to improve the diagnostic accuracy of high-grade gliomas (HGGs). The prior classification schemes relied solely on histopathologic features. The more recent classification schemes have been updated to integrate molecular features with the existing histologic criteria. Each iteration of the WHO classification refines and expands the diagnostic categories, reflecting the exponential increase in our understanding of genetic and epigenetic mechanisms driving CNS tumors. More recently, DNA methylation profiling has emerged as a formidable diagnostic tool for CNS tumor classification [1,2,3,4]. Methylation involves the addition of a methyl group to a cytosine nucleotide, predominantly occurring within CpG sites (sites wherein a cytosine nucleotide precedes a guanine nucleotide) [5]. Methylation profiles are DNA methylation patterns defined across the genome [6,7] and are fingerprints that identify the cell of origin [1,8,9,10]. These DNA methylation patterns are grouped into classifiers that are utilized for the distinct classification of tumors [1,8,9,10]. Methylation profiling is increasingly being used as an ancillary diagnostic tool to reliably diagnose otherwise equivocal high-grade glioma (HGG) tumor cases and to identify new CNS tumor entities, such as high-grade astrocytoma with piloid features (HGAP) [6,11,12].

HGAP is a described high-grade astrocytoma that is exclusively diagnosed by its distinct global DNA methylation signature [13,14]. Most commonly occurring in the posterior fossa, the morphology of HGAPs is diverse, with some overlapping features between glioblastoma and pilocytic astrocytoma [14,15]. However, characteristic histologic features of pilocytic astrocytomas, such as Rosenthal fibers, are often lacking despite the “piloid” designation [14]. HGAPs typically behave more aggressively than benign pilocytic astrocytomas but reportedly have a better prognosis compared to IDH-wild-type glioblastomas [11]. HGAPs frequently harbor alterations in the mitogen-activated protein kinase (MAPK) pathway, such as *NF1* mutations (40.4%), *FGFR1* mutations or fusions (19.1% and 14%, respectively), and *BRAF* mutations or fusions (2.3% and 18.6%, respectively), accompanied by *CDKN2A* and *ATRX* gene alterations [14]. HGAPs do not harbor *EGFR* amplification, polysomy 7/monosomy 10, and rarely harbor *TERT* promoter mutations (1.1%), which define molecular alterations for glioblastoma [6]. Along with these biological differences, which remain under investigation, divergence in treatment options between pilocytic astrocytoma and glioblastoma also remains. Intervention for this new CNS tumor entity, which lies amidst a benign and malignant tumor, is challenging. Evidence for HGAP is scant, with few reported cases and a tenuous association between treatment and patient outcomes [13,14,16].

Herein, we present a case of a 72-year-old male with a challenging HGG not elsewhere classified (NEC), showing characteristics of both HGAP and glioblastoma that did not fit neatly with any of the existing tumor entities in the 2021 WHO classification of CNS tumors, despite molecular profiling and DNA methylation analysis. We report on his response to standard-of-care glioblastoma treatment for his HGG NEC.

## 2. Detailed Case Description

**Figure 1:**
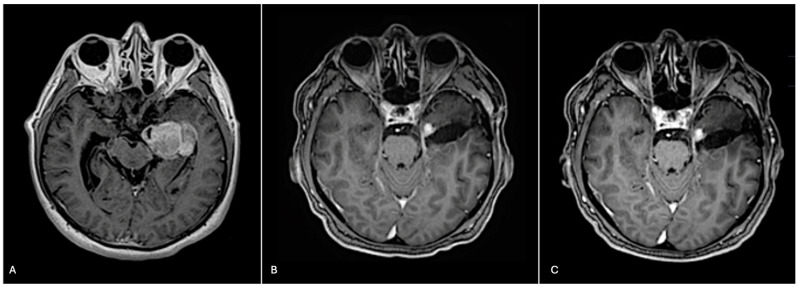
Pre-surgical, post-surgical, and post-2 cycles of temozolomide imaging. (**A**) Pre-surgical axial T1 brain MRI with contrast reveals a 4.3 cm enhancing mass. (**B**) Post-radiation axial T1 brain MRI with contrast reveals a residual 1 cm enhancing nodule at the medial aspect of the resection cavity. (**C**) Following two cycles of adjuvant temozolomide, axial T1 brain MRI with contrast reveals a stable residual 1 cm enhancing nodule at the medial aspect of the resection cavity.

**Figure 2:**
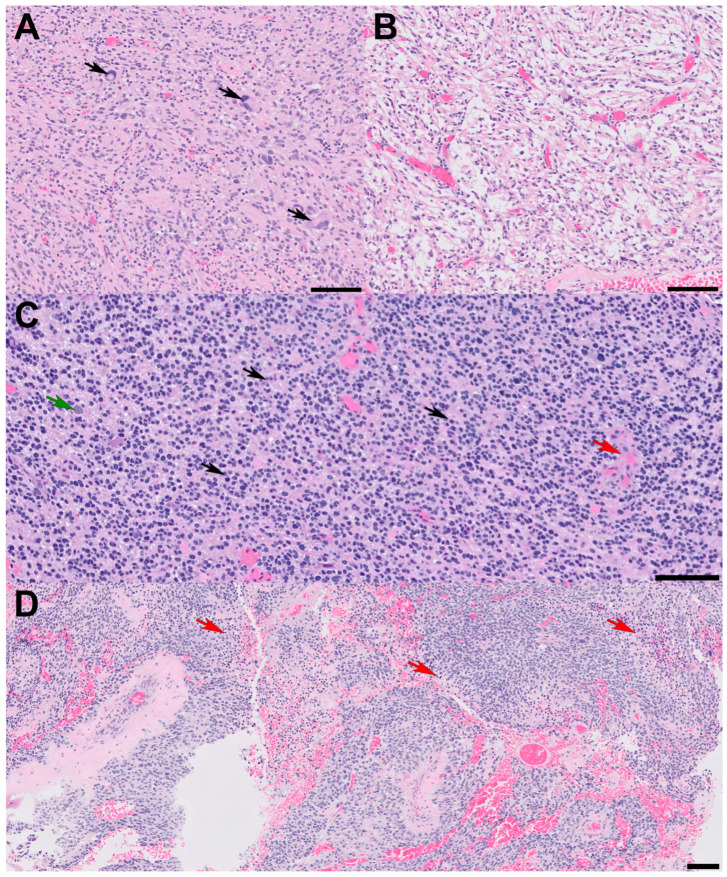
Histopathologic features. All scale bars are 100 microns. (**A**) Sections reveal glial cells with spindled and hyperchromatic nuclei with a compact growth pattern and (**B**) intervening areas of the microcystic background. Note the pleomorphic, bizarre cells with smudgy chromatin in panel (**A**) (arrows); (**C**) in other areas, tumor cells with round nuclei and scant chromatin show infiltrative growth patterns and high-grade features. Note the entrapped cortical neuron (green arrow), mitotic figures (black arrows), and incipient microvascular proliferation (red arrow); (**D**) some high-grade areas show pseudopapillary architecture due to geographic necrosis (red arrows) and viable tumor cells clinging around vessels.

**Figure 3:**
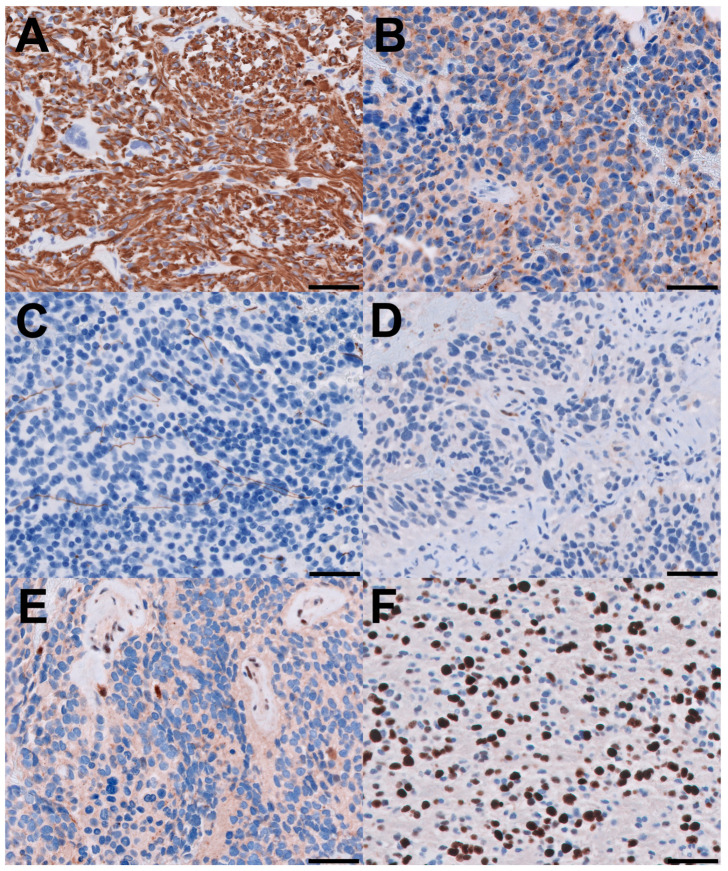
Immunohistochemical features. All scale bars are 50 microns. (**A**) Tumor cells are positive with GFAP and (**B**) weakly positive with synaptophysin; (**C**) neurofilament stain highlights entrapped axons in the high-grade areas; (**D**) p16 is essentially negative, which can be interpreted as a “loss” in tumor cells; (**E**) ATRX is lost in tumor cells in the high-grade areas and retained in endothelial cells; (**F**) Ki-67 labeling index is approximately 40% in high-grade areas.

A 72-year-old male presented with a two-day history of altered mental status and emesis secondary to a 4.3 cm enhancing mass in the left temporal region, detected on a brain MRI with and without contrast (Figure 1A). His neurological exam was notable for memory impairment. Following a subtotal resection, which left a residual 1 cm nodule at the medial aspect of the resection cavity, histopathology revealed a high-grade glial neoplasm. In some areas, tumor cells with spindled hyperchromatic nuclei showed a compact growth pattern, interspersed with intervening areas (Figure 2A,B). Rosenthal fibers and eosinophilic granular bodies were absent. Scattered in these regions were bizarre, multinucleated cells with smudgy chromatin. In other areas, tumor cells with round-to-oval nuclei and scant-to-moderate amounts of cytoplasm were embedded in a fibrillary, neuropil-like background, associated with entrapped neurons, increased mitotic activity (at least 6 mitoses per 2 mm^2^), and incipient microvascular proliferation (Figure 2C). In these infiltrative high-grade areas, geographic necrosis and viable tumor cells surrounding vasculature formed a pseudopapillary architecture (Figure 2D). Tumor cells were positive for GFAP, OLIG2, and synaptophysin (weak, diffuse in high-grade areas, focal in compact areas). Neurofilament staining was negative in compact areas and highlighted entrapped axons in infiltrative high-grade areas. The p53 stain was patchy, suggestive of the wild-type *TP53* gene. ATRX was retained in compact areas but was lost in infiltrative high-grade areas. P16 was absent from both regions. The Ki-67 labeling index was low in the compact areas but was estimated at 40% in high-grade areas (Figure 3).

**Figure 4:**
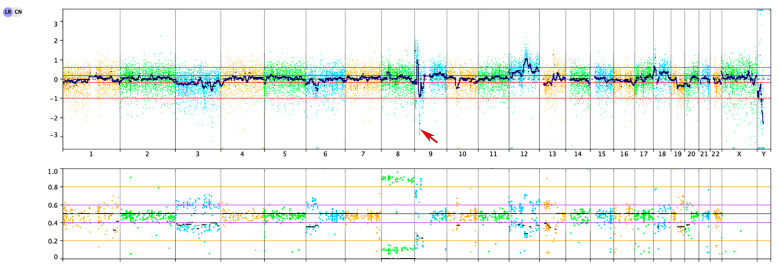
Copy number profile showed gains and losses of multiple chromosomes, and homozygous deletion of CDKN2A/CDKN2B (red arrow).

We performed targeted DNA-based next-generation sequencing (NGS) evaluating all exons of 479 cancer genes and select introns and upstream regulatory regions of 47 genes, including genes critical for glioma diagnosis, e.g., *IDH1, IDH2, EGFR, PDGFRA, MET, FGFR1, FGFR2, FGFR3, NF1, BRAF, PIK3CA, PIK3R1, PTEN, CDKN2A, CDK4, CDK6, RB1, TP53, MDM2, MDM4, H3F3A, HIST1H3B, CIC, FUBP1, ATRX,* and *TERT* (including the promoter region) [17,18]. NGS results from our case demonstrated an activating hotspot mutation in the *FGFR1* oncogene (NM_023110.2; p.N546K, variant allele frequency of 82%) and homozygous deletion of the *CDKN2A* and *CDKN2B* tumor suppressor genes on chromosome 9p21.3 in the background of additional copy number changes (Figure 4). In addition, alterations in *DNMT3A* and *TET2* were also noted, at low variant allele frequencies (7% and 5%, respectively, suggestive of clonal hematopoiesis of indeterminate potential (CHIP) [19]. No other alterations were detected, including *TERT*, *EGFR*, *PTEN*, *TP53*, *BRAF*, and *ATRX* genes, as well as polysomy 7/monosomy 10 involving the whole chromosome arms. While sequencing did not show an *ATRX* alteration, the loss of *ATRX* immunohistochemical staining (in the presence of an internal positive control) suggested either a cryptic genetic alteration or epigenetic inactivation of *ATRX.*

In addition, genome-wide methylation analysis was performed at the National Institutes of Health (NIH, Bethesda, MD, USA) after DNA extraction and bisulfite conversion (EZ DNA Methylation kit, Zymo Research, Irvine, CA, USA) using the Infinium MethylationEPIC kit (Illumina, San Diego, CA, USA). Our patient’s tumor was compared to an existing database of known CNS tumors using machine learning algorithms, including random forest and uniform manifold approximation and projection (UMAP) for dimension reduction. The class prediction was reported with a calibrated confidence score ranging from 0 to 1, with a score > 0.9, considered a “match”. This case was analyzed using versions 11b6 and 12b6 of the DKFZ Classifier (The German Cancer Research Center-Deutsches Krebsforschungszentrum, Heidelberg, Germany) and the National Cancer Institute (NCI)/Bethesda Classifier (NIH, Bethesda, MD, USA). On the Heidelberg/DKFZ classifiers, versions 11b6 and 12b6, this tumor matched to methylation class (MC)-anaplastic astrocytoma with piloid features, which is the name this classifier uses for HGAP, with a confidence score of 0.25, and to MC-pilocytic astrocytoma with a confidence score of 0.35, respectively. These matches were considered “no match” due to low confidence scores. On the NCI/Bethesda Classifier, which uses 10 different classification algorithms, this tumor matched to MC-HGAP on 8 of the 10 classifiers with high confidence scores (ranging from 0.96 to 0.99), but it matched to MC-pilocytic astrocytoma on the remaining two classifiers and on the UMAP embedding of DNA methylation array data. While the histological and molecular features of this astrocytic glioma fulfill all of the desirable criteria for HGAP, given the lack of concordance between the classifiers, an integrated diagnosis of HGG and NEC was favored. NEC is an indicator that this tumor does not conform to a specific, well-defined diagnostic entity in WHO 2021 despite adequate genetic and epigenetic testing [20].

The patient was treated with radiation and concomitant temozolomide. Post-radiation brain MRI showed a stable residual 1 cm enhancing nodule at the medial aspect of the resection cavity (Figure 1B). At the time of publication, he completed 6 cycles of adjuvant temozolomide and remains stable both clinically and radiographically (Figure 1C). Ethical guidelines set out by the Declaration of Helsinki were followed in the preparation of this report, and the patient provided written consent.

## 3. Discussion

The most recent WHO classification of CNS tumors incorporates histologic, genetic, and epigenetic features into an integrated diagnosis, which is expected to increase the accuracy and reproducibility of the diagnoses [3]. Morphologic features in this case, including infiltrative growth patterns, frequent mitoses, necrosis, and microvascular proliferation, were consistent with an aggressive astrocytoma. In the pre-integrative diagnosis era, prior to the 2016 WHO classification, these features may have been sufficient to diagnose this tumor as glioblastoma [21]. However, the tumor lacked *TERT* promoter mutation, *EGFR* amplification, and polysomy 7/monosomy 10, which are the characteristic genetic alterations of IDH-wild-type glioblastoma in adult patients [6,22,23,24]. Given the “glioblastoma IDH-wild-type” is defined by what it is lacking (i.e., lack of *IDH1* and *IDH2* hotspot mutations) rather than what it is harboring, many opportunities exist to identify new CNS tumor entities with unique genetic and epigenetic features within this HGG group. HGAP is one newly identified tumor type among HGGs, wherein a match to methylation class HGAP on DNA methylation profiling is an essential criterion [11,13,14,25].

Our case demonstrated unusual histologic features, including a relatively lower-grade region with a compact growth pattern. A pilocytic astrocytoma with high-grade progression designation was considered, given the combination of MAPK pathway alteration (*FGFR1* hotspot mutation) and ATRX loss in the high-grade areas [15]. However, the lower-grade regions did not fit neatly into characteristic pilocytic astrocytoma morphology. The presence of scattered pleomorphic cells as well as a pseudopapillary architecture also raised the possibility of high-grade glioma with pleomorphic and pseudopapillary features (HPAP), a recently proposed tumor entity with frequent *TP53* alterations and monosomy 13q [12], which were absent from our case. The histologic and molecular features also raised the possibility of HGAP [13,14,26], prompting DNA methylation profiling.

Clinical data garnered from limited existing literature for HGAP corroborated with our patient’s tumor to a limited extent. HGAP predominantly affects males with a mean age of 44 years at the time of surgery [14]. Our patient was male but was significantly older at the age of 72 at the time of surgery. Although HGAP has a predilection for the posterior fossa (62%), 26% of cases occur in the supratentorial region [14,27], as in our case where the tumor was located in the temporal lobe. An infiltrative glioblastoma-like morphology is observed in 86.7% of HGAP cases [14], substantiated by the morphology observed in our patient’s tumor. Necrosis and/or microvascular proliferation are observed in 62.3% of HGAP cases (and were present in our case) [14]. While some foci showed a compact growth pattern, Rosenthal fibers and classic pilocytic astrocytoma morphology were absent, keeping with the lack of piloid features in the majority of HGAPs [11,12,14]. Mitotic activity ranges from 0 to 6 mitoses/mm^2^ in HGAP tumors [14], which was validated by the mitotic count of 6 per HPF in our patient’s tumor. The Ki-67 proliferative index of HGAP ranges from 1 to 30%, but the Ki-67 proliferation index in our patient’s tumor was significantly elevated at 40% [14].

DNA methylation profiling was contributory, but insufficient to establish a definitive diagnosis in our case. While some of the classification algorithms used in the NCI/Bethesda Classifier matched to the methylation class “HGAP”, others, including UMAP embedding, matched to the methylation class “pilocytic astrocytoma”, while the DKFZ classifier resulted in a no-match to “anaplastic pilocytic”. Collectively, the lack of concordance and a “no match” on DNA methylation profiling yielded a final descriptive integrated diagnosis of HGG NEC.

The recent discovery of HGAP highlights the role of DNA methylation profiling in uncovering new CNS tumor entities [12,14]. Our case may represent either a new emerging CNS tumor type or subtype that has yet to be defined, or more likely, it is an unusual variant that has yet to be incorporated into the existing databases [20]. Nevertheless, using “an astrocytic glioma with DNA methylation profile of HGAP” as the sole diagnostic criterion of HGAP is not fully integrative of histologic, genetic, and epigenetic features of a tumor. Furthermore, it poses challenges to issue a specific diagnosis in cases such as this one. Accordingly, refraining from forcing a diagnosis is imperative. To our knowledge, there is only one other reported case of a suspected HGAP, with no match on DNA methylation profiling resulting in a diagnosis of HGG NEC, but treatment was not reported and remains a knowledge gap to be explored [28]. Additional cases with similar clinical, histologic, molecular, and epigenetic features are required for confirmation. Nonetheless, substantial data remain to be uncovered for HGAP, including its affiliation with pilocytic astrocytoma or glioblastoma and therapeutic options (Table 1).

Treatment for HGGs that do not conform to existing tumor entities in the WHO classification is challenging and remains a knowledge gap in literature today [14]. While standard-of-care treatment exists for glioblastoma, treatment for HGAP has not been established [29]. Treatments with the Stupp regimen, neurosurgical resection, radiation therapy, temozolomide, and targeted therapy have been reported with minimal evidence to support efficacy and with unreported long-term patient outcomes [13,14,16]. Targeted therapy, including *FGFR* inhibition, is an emerging treatment intervention for tumors with these mutations or fusions [31,32]. Preliminary evidence shows that single-agent FGFR inhibitors in recurrent gliomas with *FGFR1* mutations can achieve durable response [31]. However, our patient’s activating *FGFR1* p.N546K mutation has been reported to carry therapeutic resistance to FGFR inhibitors [33]. The lack of uniform treatment options for the genetically distinct HGAP and the lack of data on treatment for an unmatched HGG NEC posed challenges for treatment decision-making in our case. Although our patient’s tumor was not classified as either HGAP or glioblastoma, both have a poor prognosis and arguably necessitate aggressive therapy [13,34,35]. Our patient was treated with standard-of-care chemoradiation following the Stupp regimen [34]. At the time of publication, he remains clinically and radiographically stable fifteen months from the initial diagnosis.

Our case provides insights into the diagnostic challenges associated with discordant histological, molecular, and methylation results for individual patients. Our case contributes to the growing body of evidence for the treatment of HGG NEC. Further investigations are needed to better understand the clinical behavior and treatment of HGAP and to validate if this applies to our exceptional case and other similar cases [13,36,37]. As recognition and understanding of HGG NEC and HGAP improve, the treatment paradigm may also begin to diverge from standard-of-care glioblastoma treatment.

## 4. Conclusions

DNA methylation profiling is a powerful tool for uncovering new CNS tumor types and subtypes and can be used as an ancillary diagnostic tool to accurately classify challenging cases. Nevertheless, unusual cases that do not fit into established diagnostic classes exist. While some of these cases may potentially represent novel unidentified CNS tumor types, certainly all of them pose challenges in treatment decision-making for individual patients. Future investigations should be directed at understanding the therapeutic options and their associated patient outcomes for newly defined high-grade glioma entities.

## Figures and Tables

**Table 1:** High-grade astrocytoma with piloid features: confirmed or suspected reported cases.

Author/Year	Age/Sex	Location	Molecular Alterations	Surgery (s)	RT (Gy)	PFS (m)	OS (m)
Yuen et al., 2024	72/M	Temporal lobe	*CDKN2A*/B homozygous deletion *FGFR1* p.N546K ATRX loss (on IHC) *BRAF, NF1, KRAS, IDH1, IDH2, H3-3A, TERT, EGFR, PTEN, PDGFRA*–wild-type	NTR	60	15	alive at 15 m follow-up
Bender et al., 2021 [13]	71/M	Spinal cord	*CDKN2A*/B deletion ATRX loss (on IHC) *FGFR1* complex rearrangement *IDH* 1/2 wild-type *MGMT* unmethylated	STR → GTR	50.4	3.6	alive at 14.6 m follow-up
	49/F	Pons Cerebellar peduncle	*CDKN2A*/B deletion ATRX loss (on IHC) IDH1 R132H IHC negative *MGMT* unmethylated	biopsy	54	7.6	9.1
	67/M	Spinal cord	*CDKN2A*/B deletion ATRX loss (on IHC) *IDH* 1/2 wild-type *MGMT* unmethylated	STR	n/a	n/a	n/a
	53/M	Brainstem	*CDKN2A*/B deletion ATRX retained (on IHC) *IDH* 1/2 wild-type *H3-3A* wild-type *BRAF* V600 hotspot-wild-type *MGMT* methylated	STR	54	n/a	18.6
	47/M	Mesencephalon—diencephalon	NF1 syndrome *CDKN2A*/B deletion ATRX loss (on IHC) *MGMT* unmethylated	bx	n/a	n/a	1.8
	44/M	Parieto- occipital	*NF1* mutation in the setting of NF1 syndrome *CDKN2A*/B deletion ATRX retained (on IHC) *IDH1* R132H IHC negative H3 K27M IHC negative *BRAF* V600 wild-type, *MGMT* methylated	STR → STR	59.2	5.4	14.8
Cimino et al., 2023 [14] (*n* = 144)	43 (mean)/ F (*n* = 59), M (*n* = 85)	Posterior fossa (81/130, 62%) Supratentorial (34/130, 26%) Spinal Cord (13/130, 10%)	*CDKN2A* deletion (84.1%) ATRX mutation or loss on IHC (58.6%) *NF1* alterations (40.4%) *FGFR1* alterations (33.1%) *BRAF* alterations (20.9%) *KRAS* (1.1%)	n/a	n/a	n/a	n/a
Reinhardt et al., 2018 [11] (*n* = 102)	41.5 (median)/ F (*n* = 40), M (*n* = 43)	Posterior fossa (74%)	*CDKN2A*/B deletion (66/83, 80%) ATRX mutations/loss on IHC (33/74, 45%) *NF1* alterations (20/67, 30%) *BRAF* fusion (15/74, 20%) *FGFR1* alterations (12/64, 19%) *KRAS* mutation (2/64, 3%) *IDH* 1/2 wild-type (100%) *MGMT* methylated (38/83, 46%)	n/a	n/a	n/a	n/a
Nawa et al., 2024 [29]	34/M	Cerebellum/pons	*CDKN2A/B* homozygous deletion	STR	54	16	n/a
	37/F	Cerebellum, spinal cord	*CDKN2A/B* homozygous deletion	STR	60	12	n/a
Gareton et al., 2020 [30]	8/F	Supratentorial Parietal lobe	ATRX retained (on IHC) *CDKN2A* intact *FGFR1* p.K678E *RAD50* p.R365Q *MDM2* amplification Monosomy 10q	GTR	RT dose n/a	13.4	37
Zander et al., 2024 [28]	68/M	Cerebellum	*NF1* truncating mutation *CDKN2A* deletion (likely not homozygous) ATRX retained (on IHC) *BRAF, IDH1, IDH2, H3-3A, TERT* wild-type	GTR	n/a	n/a	n/a

*ATRX* = alpha thalassemia/mental retardation syndrome X-linked; *BRAF* = B-Raf proto-oncogene; *CDKN2A* = cyclin-dependent kinase inhibitor 2A; *FGFR1* = fibroblast growth factor receptor 1, GTR = gross total resection, Gy = Gray; *H3-3A* = H3 histone family member 3A; *IDH* isocitrate dehydrogenase; m = months; *MGMT* = O6-methylguanine-DNA-methyltransferase, n/a = not available, OS = overall survival, *NF1* = neurofibromatosis type 1; *PTEN* = Phosphatase and TENsin homolog deleted on chromosome 10; PFS = progression-free survival; RT = radiotherapy; STR = subtotal resection, IHC: Immunohistochemistry.

## Data Availability

Data are unavailable due to privacy.

## Abbreviations

ATRX	alpha thalassemia/mental retardation syndrome X-linked
BRAF	B-Raf proto-oncogene
CDKN2A	cyclin-dependent kinase inhibitor 2A
CNS	central nervous system
EGFR	epidermal growth factor receptor
FGFR1	fibroblast growth factor receptor 1
GTR	gross total resection
H3-3A	H3 histone family member 3
HGAP	high-grade astrocytoma with piloid features
IDH	isocitrate dehydrogenase
HGG	high-grade gliomas
IHC	immunohistochemistry
MAPK	mitogen-activated protein kinase
MGMT	O6-methylguanine-DNA-methyltransferase
NEC	not elsewhere classified
NF1	neurofibromatosis type 1
OS	overall survival
PTEN	Phosphatase and TENsin homolog deleted on chromosome 10
RT	radiation therapy
STR	subtotal resection
TERT	telomerase reverse transcriptase
WHO	World Health Organization
